# Sustainable Carotenoid Extraction from Macroalgae: Optimizing Microwave-Assisted Extraction Using Response Surface Methodology

**DOI:** 10.3390/life14121573

**Published:** 2024-11-30

**Authors:** Andreia Lopes, Luísa Correia-Sá, Mónica Vieira, Cristina Delerue-Matos, Cristina Soares, Clara Grosso

**Affiliations:** 1 REQUIMTE/LAQV, ISEP, Polytechnic of Porto, Rua Dr. António Bernardino de Almeida 431, 4249-015 Porto, Portugal; 10180563@ess.ipp.pt (A.L.); mlsrs@isep.ipp.pt (L.C.-S.); cmm@isep.ipp.pt (C.D.-M.); 2 Chemical and Biomolecular Sciences, School of Health (ESS), Polytechnic of Porto, 4200-465 Porto, Portugal; mav@ess.ipp.pt; 3 Center for Translational Health and Medical Biotechnology Research (TBIO)/Health Research Network (RISE-Health), ESS, Polytechnic of Porto, 4200-072 Porto, Portugal

**Keywords:** seaweeds, carotenoids, microwave-assisted extraction, high-performance liquid chromatography, Box–Behnken design

## Abstract

This study aimed at optimizing carotenoid extraction using the macroalga *Himanthalia elongata* (L.) S.F.Gray as a model. Firstly, traditional extraction procedures were employed, using various solvents and temperatures to enhance the extraction conditions. Once the most effective extraction conditions were identified, the study transitioned to a more efficient and environmentally friendly approach, microwave-assisted extraction (MAE). By applying a three-parameter (solid-to-solvent ratio, temperature, and time) Box–Behnken design, the optimal extraction conditions were found to be a solid-to-solvent ratio of 1/13.6 g/mL at 60 °C for 15 min. Under these conditions, the predicted and experimental carotenoid contents were 2.94 and 2.12 µg/mL, respectively. Furthermore, an HPLC-DAD method was developed and validated for the characterization of carotenoids. β-Carotene was the predominant carotenoid in *H. elongata*, alongside fucoxanthin. The optimized MAE method was applied to other seaweeds, including *Fucus vesiculosus* L., *Codium tomentosum* Stackhouse, *Gracilaria gracilis* (Stackhouse) Steentoft, L.M.Irvine & Farnham, and *Eiseinia bicyclis* (Kjellman) Setchell. Among all, *F. vesiculosus* exhibited the highest carotenoid content compared to the others. This study concludes that MAE under optimized conditions is an effective and sustainable approach for carotenoid extraction, providing significant yields of bioactive compounds such as β-carotene and fucoxanthin, which have promising applications in enhancing human health and nutrition.

## 1. Introduction

Seaweeds are photosynthetic organisms that live in subaquatic environments with various shapes, colours, and morphologies [1,2]. They have an attractively healthy nutritional composition, in terms of protein, vitamins, and minerals, allied to low-calorie content [3]. Besides, seaweeds are a rich source of structurally diversified bioactive compounds such as peptides, lectins, carotenoids, polysaccharides, fatty acids, phenolic compounds, and phytosterols [4]. Due to these bioactive compounds, macroalgae present a wide variety of health-promoting properties such as antioxidant, antiviral, antifungal, antibacterial, antiproliferative, anti-inflammatory, neuroprotective, anti-adipogenesis, antidiabetic, and anti-obesity properties, reducing blood pressure effects, and helping digestion [5,6,7]. Recently, algae have been increasingly incorporated into dietary habits worldwide because of the reported properties mentioned above and their nutritional value [3,5]. Additionally, various applications in different areas, like the textile, pharmaceutical, biofuel, and food industries, have been exploited [4,5,8].

Being a natural photosynthetic resource, seaweeds are characterised by pigments, such as chlorophylls and carotenoids. Carotenoids, in particular, provide a wide range of bioactivities, mainly due to one major carotenoid, fucoxanthin [9]. Fucoxanthin is predominant in brown algae, corresponding to about 10% of total carotenoid content, and has many beneficial properties as a radical scavenger with anti-obesity and cancer benefits like antiproliferative, anti-inflammatory, and apoptotic effects [10]. Lately, its potential antioxidant activity has been investigated, and the protector effect of fucoxanthin has been reported against oxidative stress and DNA damage derived from UV radiation [10,11]. Furthermore, some carotenoids, such as β-carotene, have vitamin A precursor properties and can even protect against macular degeneration [1]. Besides, β-carotene has a strong antioxidant activity because of its conjugated double bonds, which allow the quenching of free radicals. For this reason, this pigment is frequently used and easily incorporated into cosmetics or food additives [10,12].

The extraction of bioactive compounds from algae is usually achieved through maceration. The use of an appropriate temperature to optimise the extraction, and the choice of a suitable solvent, either organic or aqueous, according to the structure of the carotenoids, are important factors to consider [13,14,15,16]. Carotenoids have, in the majority, high hydrophobicity and require non-polar solvents, like acetone, dichloromethane, or biphasic mixtures, or some green solvents such as ethanol, limonene, and some other mixtures with water. In contrast to the efficiency of this method, there is a significant disadvantage since this process consumes much time and may include many steps, leading to a high consumption of solvents to obtain a final yield that may not compensate for the resources used [13]. On the other hand, microwave-assisted extraction (MAE) is an efficient solid-liquid extraction (SLE) method with a low initial cost, used mainly to extract bioactive compounds in algae such as polysaccharides, essential oils, and carotenoids [17]. This method combines microwaves and traditional solvent extraction. It involves non-ionising radiation that will cause changes in the cell structure, disrupt the cell membranes, and promote the bioactive compounds’ exit [18,19]. The fundamental parameters that must be optimised when extracting carotenoids from seaweeds using MAE are time, temperature, the algae/solvent ratio, and potency, among other features [10]. MAE has been reported to be more efficient than ultrasound-assisted extraction, enzyme-assisted extraction, pulsed electric field extraction, and pressurised liquid extraction, among other techniques [12,13]. However, some disadvantages cannot be neglected, such as thermal degradation, which can cause cis–trans isomerisation of carotenoids, and the scale-up of the process being difficult to achieve [12].

A combination of extraction and analytical methods, such as MAE and high-performance liquid chromatography coupled to a diode array detector (HPLC-DAD) was applied in the current study to optimise the extraction and characterisation of the carotenoid profile of *Himanthalia elongata* (L.) S.F. Gray, a macroalga recognised as containing a significant amount of carotenoids [13,20,21]. After extraction optimisation using a Box–Benhken design (BBD), the optimal extraction conditions were also applied to four other species of macroalgae (*Codium tomentosum* Stackhouse, *Eisenia bicyclis* (Kjellman) Setchell, *Fucus vesiculosus* L., and *Gracilaria gracilis* (Stackhouse) Steentoft, L.M.Irvine & Farnham) available in the Portuguese market. BBD is a response surface methodology (RSM) tool widely used to optimise extraction conditions [22,23]. This design correlates at least three independent variables and combines their information to achieve a theoretical optimal point. Different responses can be optimised, and a quadratic or another model must be fitted with statistical significance to the experimental results. As an outcome of the fitting, a mathematical equation is given by the software, and later, based on that equation, a desirability function can be used for optimisation purposes [23].

This study aims to address the inefficiencies and environmental impact of traditional carotenoid extraction methods by optimizing an MAE process using *H. elongata* as a model species. Using RSM, this research identifies the ideal conditions for maximizing carotenoid yields while ensuring sustainability. Additionally, a validated HPLC-DAD method is developed to accurately characterize carotenoid profiles.

Beyond the technical optimization, this study evaluates the potential of seaweeds as functional foods to enhance carotenoid intake, particularly β-carotene and lutein, which are vital for eye health. This assessment involves analysing the carotenoid content of seaweed extracts obtained under optimal conditions and comparing the yields and profiles across different species. The findings were contextualized with dietary recommendations to determine the extent to which seaweed consumption could meet daily carotenoid requirements. This dual focus on sustainable extraction and dietary relevance highlights the broader applications of seaweeds in nutrition, health, and industry.

## 2. Materials and Methods

### 2.1. Samples and Pre-Treatment

*H. elongata* was obtained from Algamar, with a mean moisture content of 8.82%. The moisture content was determined using a thermogravimetric moisture analyser (Kern DAB 100-3, Balingen, Germany). The remaining macroalgae samples were purchased from Algaplus (*F. vesiculosus, G. gracilis*, and *C. tomentosum*) and Algamar (*E. bicyclis*). The mean moisture content of these macroalgae samples was 7.11% (*F. vesiculosus*), 9.56% (*G. gracilis*), 8.91% (*C. tomentosum*), and 7.99% (*E. bicyclis*). The moisture analysis was performed in triplicate.

Before extraction and analysis, *H. elongata* was hydrated with salted water (35 g/L NaCl) for 5 min, then the seaweed was drained for the same amount of time and washed in deionised water for a few minutes to eliminate the excess salt. The seaweed was then dehydrated for about 14–18 h in a food dehydrator (Excalibur, model 4926T, Dublin, Ireland) at 41 °C before grinding. A Moullinex A320 (Écully, France) was used to grind and homogenise the dehydrated samples that were then sieved to a 1–2 mm particle size with a vibratory sieve shaker (Retsch, Haan, Germany). The same procedure was applied to the remaining algae (*C. tomentosum, E. bicyclis, F. vesiculosus*, and *G. gracilis*).

### 2.2. Extraction of Carotenoids

According to the literature review, ethanol, acetone, and mixtures containing these solvents were the most commonly elected solvents for carotenoid extraction [10,17,18,19,24,25]. Thus, the solvents selected for extraction were 100% acetone (Sigma-Aldrich, Steinhein, Germany), 100% ethanol absolute (Carlo Erba, Val de Reuil, France), and mixtures of these solvents with water: 90% acetone, 90% ethanol, and 50% ethanol.

The conventional extraction protocol for carotenoids from seaweeds was adapted from previous studies [26,27]. Briefly, 500 mg of seaweed was hydrated with ultrapure water (resistivity of 18.2 MΩ·cm at 25 °C, produced using a Simplicity 185 system from Millipore, Molsheim, France) for 5 min, and after discarding excess water, the chosen solvent was added according to the desired solid/solvent ratios 1/5; 1/10; 1/15 g/mL. The mixture was kept in constant agitation for 30 min, at 600 rpm, at room temperature (25 ± 2 °C). Then, the supernatant was separated, and the procedure was repeated on the same matrix. Finally, the supernatants were mixed and centrifuged for 5 min, and the total carotenoid amount was subsequently determined spectrophotometrically. The process was also conducted using a higher temperature (40 °C). Each seaweed sample was placed in an Erlenmeyer flask covered with aluminium foil to minimize solvent evaporation at elevated temperatures. The flasks were set on a hot plate magnetic stirrer (VWR, Model VMS-C7, Alfragide, Portugal) to maintain the desired temperature and ensure constant stirring at 200 rpm.

After the extractions, the solvent was evaporated with a rotary evaporator (Büchi, Flawil, Switzerland), and the extracts were dried in the desiccator until constant weight. Finally, the dried extracts were redissolved with methanol (VWR., Gliwice, Poland) before further analysis. Extractions were performed in triplicate (*n* = 3).

### 2.3. Spectrophotometric Quantification of Carotenoids and Chlorophylls

The spectrophotometric measurement of chlorophylls and carotenoids was performed using 96-well plates in a Synergy HT W/TRF multimode microplate reader (BioTek Instruments, Winooski, VT, USA) using Gen5 2.0 software (BioTek Instruments) from 300 to 800 nm. The total carotenoid content, as well as total chlorophyll a, b, and c content, were calculated by the following equations [28]:

$$Carotenoids\;(µg/mL)=4\times \left({Abs}_{480}-{Abs}_{750}\right)$$

$$\begin{array}{l} Chl\;a\;\left(µg/mL\right)=-2.0780\times \left({Abs}_{632}-{Abs}_{750}\right)-6.5079\times \left({Abs}_{652}-{Abs}_{750}\right)\\ \;\;\;\;\;\;\;+16.2127\times \left({Abs}_{665}-{Abs}_{750}\right)-2.1372\times \left({Abs}_{696}-{Abs}_{750}\right) \end{array}$$

$$\begin{array}{l} Chl\;b\;\left(µg/mL\right)=-2.9450\times \left({Abs}_{632}-{Abs}_{750}\right)+32.1228\times \left({Abs}_{652}-{Abs}_{750}\right)\\ \;\;\;\;\;\;\;-13.8255\times \left({Abs}_{665}-{Abs}_{750}\right)-3.0097\times \left({Abs}_{696}-{Abs}_{750}\right) \end{array}$$

$$\begin{array}{l} Chl\;c\;\left(µg/mL\right)=34.0115\times \left({Abs}_{632}-{Abs}_{750}\right)-12.7873\times \left({Abs}_{652}-{Abs}_{750}\right)\\ \;\;\;\;\;\;\;-1.4489\times \left({Abs}_{665}-{Abs}_{750}\right)-2.5812\times \left({Abs}_{696}-{Abs}_{750}\right) \end{array}$$

These equations were applied to determine the carotenoid and chlorophyll content in the extracts obtained from the preliminary conventional extractions, and the extraction conditions allowing the highest concentration of total carotenoid content were considered for the BBD to perform MAE. The absorbance was generally below 1, and the samples were diluted whenever necessary. Each determination was performed in triplicate (*n* = 3). All extraction conditions were compared to the condition that achieved the highest concentration of carotenoids and chlorophylls using ANOVA followed by Dunnett’s post-test in GraphPad Prism 8.0.1. Statistical significance is represented as follows: (****, *p* < 0.0001; ***, *p* < 0.001; **, *p* < 0.01; *, *p* < 0.05; n.s., *p* > 0.05).

### 2.4. Response Surface Methodology (RSM) for MAE

The parameters considered for BBD were time, temperature, and solid/solvent ratio, which varied between 5–25 min, 20–60 °C, and ratios of 1/5 to 1/15 g/mL, respectively according to Table 1. In total, 15 runs (with 3 central points) were performed. Total carotenoid content (µg/mL) was considered the dependent variable.

The modelling and the optimisation processes for *H. elongata* were performed using the software Design Expert 11 (Stat-Ease Inc., Minneapolis, MN, USA). After obtaining the optimal extraction point, an MAE with the optimum conditions was performed for the remaining macroalgae species.

### 2.5. Microwave-Assisted Extraction (MAE)

Three parameters defined through RSM—time, temperature, and solid/solvent ratio—were tested according to Table 1. For MAE, a MARS-X 1500 W (Microwave Accelerated Reaction System for Extraction and Digestion, C.E.M., Mathews, NC, USA) was used, with 14 polytetrafluoroethylene (PTFE) digestion vessels and temperature (Probe RTP—300 Plus, C.E.M.; ±3 °C) and pressure (Digital Pressure Gauge ESP 1500 Plus, C.E.M.; ±10 psi) control sensors. Around 500 mg of homogenised sample was weighed in a microwave PTFE vessel, and 5 mL of ultrapure water was added to hydrate the samples for 5 min. After removing excess water, the solvent—ethanol/water 90:10 (*v*/*v*)—was added according to the desired solid/solvent ratio. The MAE equipment conditions for the optimal point are represented in Table 2.

After the extractions, the solvent was evaporated at reduced pressure, as described previously. Afterwards, the dried extracts were dissolved with 2 mL of methanol for total carotenoid and chlorophyll content determination.

Each run was performed in triplicate (*n* = 3). Conditions at the optimum point were also used to extract carotenoids from the other macroalgae species (*C. tomentosum, E. bicyclis, F. vesiculosus*, and *G. gracilis*). The total carotenoid content of the 15 runs of the BBD plus the extractions of the other seaweeds were assessed using the equations reported in Section 2.4.

### 2.6. HPLC-DAD Identification of Carotenoids

Twenty microliters of each sample were analysed (*n* = 3) on an analytical HPLC unit (Shimadzu, Kyoto, Japan) consisting of a low-pressure quaternary pump (model LC-20AT), a degasser (model DGU-20A5R), an auto-sampler (model SIL-20AT), a column oven (model CTU-20AC), and a photodiode array detector (model SPD-M20A High-Performance Liquid Chromatography P.D.A. detector). Compound separation was achieved with a C30 YMC Carotenoid S-5 µm (25.0 × 0.46 cm; 5 μm particle size) column (Kyoto, Japan). The solvent system consisted of methanol (A) (Methanol Chromasolv for HPLC from Riedel-de Haën, Seelze, Germany) and methyl tert-butyl ether (MTBE) (B) (MTBE for HPLC from Carlo Erba, Val de Reuil, France) starting with 95% A, using a gradient to obtain 70% A at 30 min, 50% A at 60 min, and again 95% A at 65 min, based on the method developed by Fernandes et al. [26]. The solvent flow rate was 0.9 mL/min. Spectral data from all peaks were collected in the 200–800 nm range, and chromatograms were recorded at 450 nm. Data were processed on LabSolutions software version 5.82 (Shimadzu). The compounds were identified by comparing their retention times and UV–vis spectra with standards (β-carotene and fucoxanthin purchased from Sigma-Aldrich, Steinhein, Germany, and zeaxanthin and lutein from Extrasynthèse, Genay, France) injected in the same conditions as with the data from earlier literature [29,30,31]. External calibration curves were prepared for quantification using different concentrations (*n* = 3, each concentration) and recording peak areas at 450 nm.

## 3. Results and Discussion

### 3.1. Conventional Extraction

Carotenoid extraction from seaweeds was performed using a conventional extraction at room temperature and 40 °C. Ethanol, acetone, and mixtures of these solvents with water at different solid/solvent ratios were tested. The choice of these solvents and mixtures was based on previous reports showing that the most predominant solvents used to extract carotenoids are acetone, hexane, ethanol, and ethanol/hexane mixtures. Among the alcohols, ethanol is preferred because, besides being a low-priced product, this organic solvent helps extract the carotenoids with a hydrophobic profile and can be quickly introduced in the cosmetic and food industry [12].

Table 3 presents the total carotenoid and chlorophyll contents obtained after the conventional extraction procedure using several solid/solvent ratios (1/5; 1/10; 1/15 g/mL) and temperatures (25 °C, 40 °C) for 30 min, with the procedure being performed twice.

**Figure 1:**
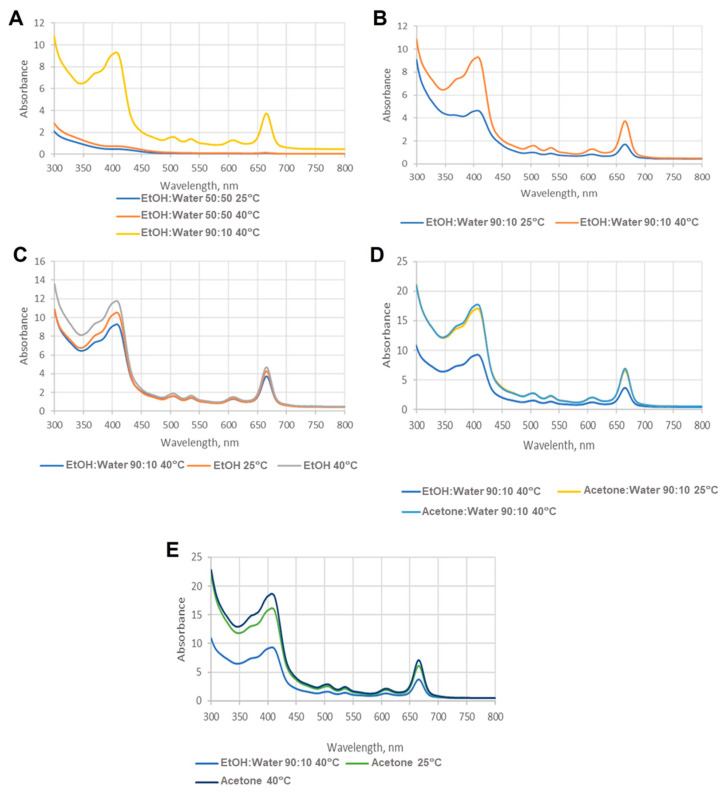
Spectra comparison of the samples, where (**A**–**E**) represent the results of the different solvents used in the conventional extraction.

Considering the parameter temperature, it is possible to infer that a higher temperature (40 °C vs. 25 °C) was a determinant factor in improving the carotenoid content extracted (Table 3, Figure 1A–E). Indeed, as observed in Figure 1, the higher the temperature, the higher the absorbance intensity in all the cases presented.

Concerning the solid/solvent ratio, it was observed that the lowest ratio (1/5 g/mL) produced the lowest carotenoid content due to the small quantity of solvent available for the extraction (Table 3) and possible saturation of the solvent [32,33,34]. Conversely, the ratio of 1/15 g/mL increased carotenoid extraction and concentration (Table 3).

As reported in the literature, the colour change on the algae residue after the extraction is due to the efficiency of the extracting solvent. The more greyish the colour, the higher the amount of pigment extracted [35,36]. All the samples extracted with acetone produced extracts with a higher carotenoid content, ranging from 0.0126 to 0.0942 µg of total carotenoids/mg of extract for solid/solvent ratios of 1/5 to 1/15 g/mL at 40 °C, respectively (Table 3). However, as it is possible to observe from Figure 1, the acetone extracts’ spectra showed a huge maximum at ≈600 nm, with these peaks corresponding to chlorophylls, revealing the lack of selectivity of acetone.

Moreover, acetone and acetone/water mixtures revealed the highest concentration not only of carotenoids but also of chlorophylls, with values ranging from 0.285 (1/10 g/mL; 25 °C) to 0.887 (1/10 g/mL; 40 °C) and 0.0556 (1/5 g/mL; 25 °C) to 1.08 (1/15 g/mL; 40 °C) µg chlorophylls/mg of extract for 90% acetone and 100% acetone, respectively (Table 3).

Despite a high extraction yield, this solvent cannot be present in food and cosmetic products, and it needs to be completely evaporated before extracts are allowed to be used in the food industry. This evaporation step means extra work and costs, which may not be acceptable to the industry [37]. Solvent mixtures based on ethanol show a good extraction yield at 90%, having good carotenoid content (up to 0.0353 µg carotenoids/mg of extract) and lower chlorophyll content (up to 0.451 µg chlorophylls/mg of extract) when compared with 100% ethanol (with slightly higher carotenoid content but also with a higher amount of chlorophylls) and 50% ethanol (with lower carotenoid and chlorophyll content) (Table 3). Besides, the higher temperature increased the carotenoid content extracted.

In another study, a conventional extraction of carotenoids and chlorophylls from the microalga *Chlorella vulgaris* Beijerinck was performed with acetone and ethanol, among other solvent mixtures. The results showed that with 90% ethanol, a higher yield (30%) was obtained compared to the other solvents [16]. However, the extraction conditions were not similar to those used in the present study. Babadi et al. [38] showed that acetone was less selective (carotenoids = 2.01 mg/g algae d.w. and chlorophylls = 10.1 mg/g algae d.w.) than dimethyl ether in extracting carotenoids from the green microalga *Chlorococcum humicola* (Naegeli) Rabenhorst. Compared with the present study, where similar results were obtained, this can be explained because acetone is a less polar solvent than water and ethanol, which can increase the extraction of carotenoids and chlorophylls, although carotenoids are less polar than chlorophylls [38].

So, to proceed with the study, the solvent elected was 90% ethanol because despite revealing a lower extraction yield compared to 100% acetone, it is a solvent that is largely used and easily accepted in the food and pharmaceutical industries and presents a higher selectivity to carotenoids when compared with acetone [37,39].

### 3.2. Carotenoid Extraction Through Microwave-Assisted Extraction (MAE)

The MAE conditions used in this study considered the BBD matrix presented in Table 1, with three central points (15 min, 40 °C, 1/10 g/mL solid/solvent ratio, runs 13–15). The solvent selected to perform MAE was 90% ethanol, and the obtained results for the carotenoid content per extracted mass are presented in Table 4.

**Figure 2:**
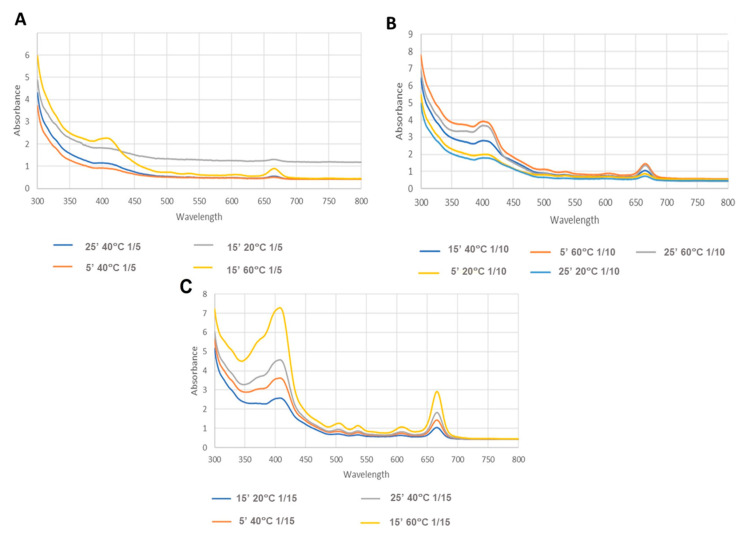
Spectra of MAE extracts, where (**A**–**C**) represent the results of the different solid/solvent ratios: (**A**)—1/5; (**B**)—1/10; (**C**)—1/15 g/mL.

**Figure 3:**
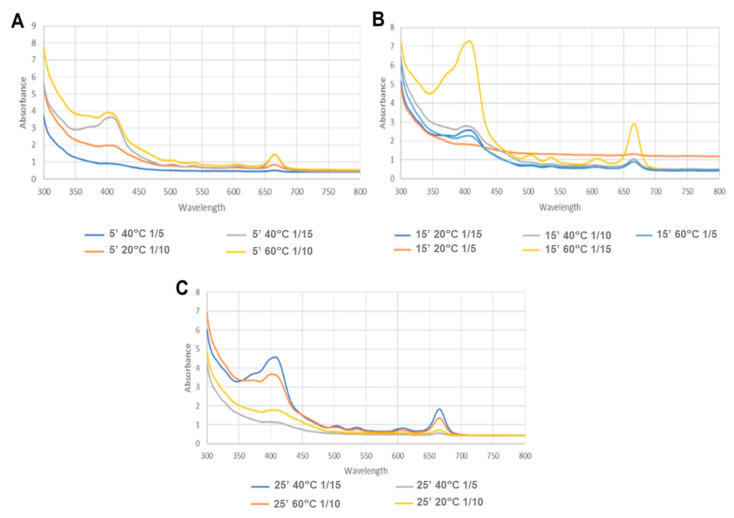
Spectra of the MAE extracts, where (**A**–**C**) represent the results of the different times, namely (**A**)—5 min; (**B**)—15min; (**C**)—25 min.

**Figure 4:**
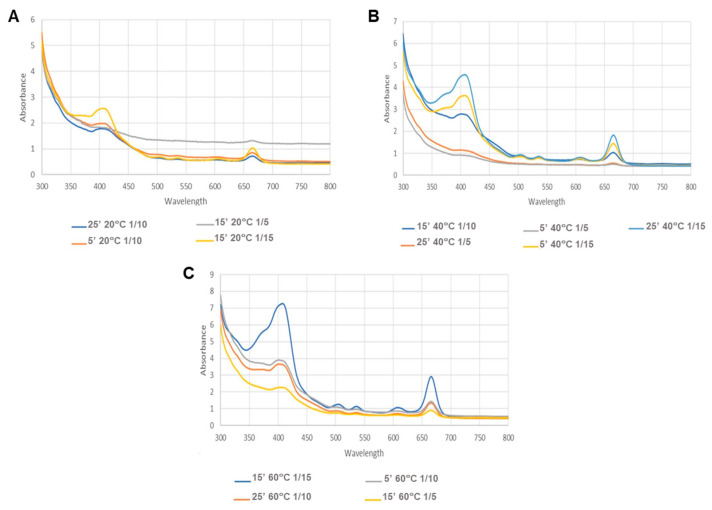
Spectra of the MAE, where (**A**–**C**) represent the results of the different temperatures, namely (**A**)—20 °C; (**B**)—40 °C; (**C**)—60 °C.

Analysing the results obtained for MAE (Table 4, Figure 2, Figure 3 and Figure 4), it is evident that the variable solid/solvent ratio greatly influences the extraction.

A lower carotenoid content was obtained when using a minor solid/solvent ratio (1/5 g/mL). The largest ratio (1/15 g/mL) showed a higher absorbance, which can also be seen in Figure 2, resulting in a higher carotenoid content of approximately 0.0634 µg/mg of extract (Table 4).

Besides the ratio, temperature also showed itself to be a significant parameter; that is, at higher temperatures, the content of carotenoids was increased independently of the ratio or time. The better temperature for extraction was 60 °C, showing a higher absorbance in the 450 nm region (Figure 4). On the other hand, the factor time did not have an impact on the extraction.

Some studies show that MAE can be an efficient method for carotenoid extraction. However, the thermal degradation caused by high temperatures can damage the carotenoids, provoking their degradation and *cis* and *trans* isomerisation. However, this problem can be easily solved by lowering the temperature [40]. Carotenoid extraction from the green alga *Cladophora glomerata* (L.) Kützing showed good results with MAE extraction with ethanol, having a concentration of total carotenoids like that obtained in the present study. Among all the extraction methods tested in extracting the pigments from green algae, MAE (60 min, 40 °C) yielded a higher content [41]. Sarma [42] tested different solvents (acetone, hexane, methanol, hexane/acetone (1:1), hexane/ethanol (7:3)) for MAE of *Chlorella* biomass. The highest carotenoid yield (0.061 mg/g sample d.w.) was achieved with the extraction performed with acetone at 50–52 °C, 1.69 min, 130 W, and a ratio of 1 g/20 mL.

### 3.3. Response Surface Methodology (RSM)

The information fed into the BBD mathematical model came from calculating the total carotenoid content (µg/mL) through the equation reported in the material and methods section (Section 2.4). All runs were performed by MAE because it is a more efficient method for extracting these pigments.

With the complete ANOVA quadratic model, the calculated coefficients of determination (*R*^2^), adjusted *R*^2^, predicted *R*^2^, and adeq precision were, respectively, 0.9489, 0.8570, 0.2436, and 10.9115. The model was significant (*p* = 0.0096), and the lack-of-fit was not significant (*p* = 0.1272). The second-order polynomial equation retrieved from the simulation to describe the effect of the independent variables and their interactions on the response using the BBD was as follows:

$$\begin{array}{l} Carotenoid\;content\\ \;\;\;\;\;=-2.05-0.0630X1+0.5667X2+0.7510X3-0.0187X1X2-0.0227X1X3\\ \;\;\;\;\;+0.1363X2X3-0.0937{X1}^{2}+0.1408{X2}^{2}-0.4662{X3}^{2} \end{array}$$

To optimise the results and improve the predicted *R*^2^, a further simulation was carried out by applying the ANOVA reduced quadratic model (*p* < 0.0001) since only parameters X2 (*p* < 0.0001), X3 (*p* < 0.0001) and X3^2^ (*p* = 0.0016) were found significant. As a result, the values of correlation increased: *R*^2^ = 0.9229, adjusted *R*^2^ = 0.9018, predicted *R*^2^ = 0.8402, adeq precision = 20.8228, and the lack-of-fit was not significant (*p* = 0.2336). The equation of the reduced model in terms of actual factors is as follows:

$$Carotenoid\;content=2.08+0.5668X2+0.7510X3-0.4696{X3}^{2}$$

**Figure 5:**
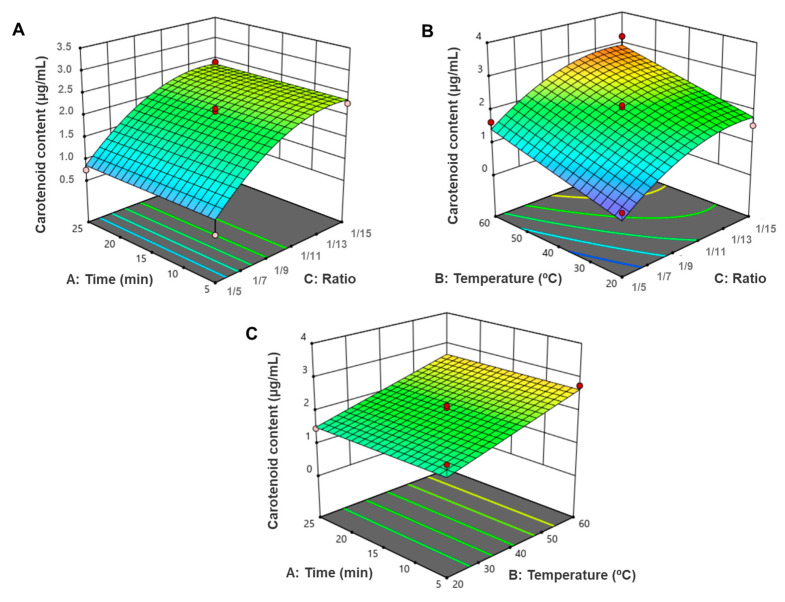
Response methodology surface 3D graphic of the reduced quadratic model with three central points, where the key conditions were (**A**) time vs. ratio; (**B**) temperature vs. ratio; (**C**) time vs. temperature.

With the reduced model, the predicted and actual values were much closer, revealing a better accuracy, as is possible to observe in Figure S1 and Table 4. Moreover, the 3D response surface graphic revealed that the major significant parameters were the ratio and the temperature, as is displayed in Figure 5.

After obtaining the model with the best fit for the experimental data, an optimisation tool, Derringer’s desirability function, was used to simulate the parameter values that would maximise the carotenoid content. As a result, the highest desirability (1.000) and predicted carotenoid content (2.942 µg/mL) were achieved while keeping the temperature at the maximum (60 °C) and the solid/solvent ratio also close to the highest tested (1/13.6 g/mL). However, time conditions showed little influence since the same desirability and carotenoid content were obtained for 5, 15, and 25 min. Therefore, the conditions selected for further studies were time = 15 min, solid/solvent ratio = 1/13.6 g/mL, and temperature = 60 °C.

A previous study focused on the subcritical fluid extraction of carotenoids from seaweeds, using this statistical method, showed that the temperature, pressure, and co-solvent were the parameters that had a significant impact on the extraction yield of carotenoids [43]. Another study aimed to optimise the supercritical CO_2_ extraction of carotenoids from the brown seaweed *L. japonica*. It was determined that the co-solvent flow rate and the temperature were crucial factors in increasing the extraction yield, and the pressure was also a factor that had great significance [44]. Vieira et al. [45] optimised the carotenoid extraction from a brown alga, *Sargassum muticum* (Yendo) Fensholt, by RSM, finding that the solid/liquid ratio was the most important significant variable, and Xiao et al. [46] studied the influence of microwave power, solid/solvent ratio, irradiation time, and extraction temperature on MAE of fucoxanthin from different brown macroalgae. The authors found that only microwave power was not a statistically significant factor. Comparing the studies referred to above, it can be concluded that the present study has crucial parameters that are similar to all studies, namely the solid/solvent ratio and the temperature, although the extraction method was not always the same as those used in the reported studies.

After determining the optimisation point to obtain the greatest amount of total carotenoids, MAE extraction from *C. tomentosum*, *F. vesiculosus*, *E. bicyclis*, *G. gracilis*, and *H. elongata* was performed using the optimised extraction conditions. Total carotenoid content was determined based on the equation reported in Section 2.4, showing that *C. tomentosum* (3.76 ± 0.28 µg/mL; 0.143 ± 0.023 µg carotenoids/mg extract dry weight (d.w.)), *F. vesiculosus* (3.85 ± 0.23 µg/mL; 0.153 ± 0.021 µg carotenoids/mg extract d.w.), and *E. bicyclis* (4.20 ± 0.35 µg/mL; 0.127 ± 0.017 µg carotenoids/mg extract d.w.) are richer in carotenoids than *H. elongata* (2.12 ± 0.17 µg/mL; 0.0428 ± 0.0051 µg carotenoids/mg extract d.w.) and *G. gracilis* (1.19 ± 0.16 µg/mL; 0.0574 ± 0.0157 µg carotenoids/mg extract d.w.) (Table 5).

Regarding the alga *E. bicyclis*, a quantity of 0.42 mg of fucoxanthin/g was reported in the literature [47] using a pressurised solvent extraction (PSE) extraction method with the following conditions: 103.4 bar; 110 °C; 5 min; 2 g of algae; EtOH 90%; static. The value for the extract under study is lower than the value obtained. However, it is essential to consider that the biochemical profile of seaweeds vary even in the same species, with influencing factors including the geographical location and time of the year in which the harvest was performed, the growth stage, the algal tissue sampled, the sampling site, and the storage time and conditions [48]. *C. tomentosum* was reported to have about 0.01 mg of total carotenoids/g algae f.w. (fresh weight) [49] in which acetone was used as the extracting solvent. *H. elongata* was shown to have a carotenoid content of 2.3 µg/g algae [24] using methanol as the solvent for conventional extraction. Bianchi et al. [50] quantified four carotenoids in *F. vesiculosus*, amounting to 269.3 µg/g d.w. To the best of our knowledge, the carotenoid content of *G. gracilis* was not found in the literature, although an unsaponified fraction of the total lipids extracted from *G. gracilis*, which includes waxes, sterols, fat-soluble vitamins, and carotenoids, was found to correspond between 0.15–0.35 % d.w. [51].

### 3.4. Carotenoid Profiles of MAE Extracts

Four different carotenoids were monitored in the MAE extracts obtained at the optimal conditions: fucoxanthin (ʎ_max_ = 448 nm), lutein (ʎ_max_ = 422, 444, 472 nm), zeaxanthin (ʎ_max_ = 428, 450, 476 nm), and β-carotene (ʎ_max_ = 452, 477 nm). The HPLC-DAD method was validated for these standards based on linearity, the limit of detection (LOD), quantification (LOQ), precision, and accuracy. Linearity and sensitivity were evaluated from the coefficients of determination (*R*^2^) of the regression curves obtained for each standard and from the values of LOD and LOQ, respectively. As can be seen in Table 6, the HPLC-DAD method showed good linearity and sensitivity, with *R*^2^ between 0.9928 and 0.9998 and low LODs and LOQs.

Furthermore, intraday and interday precision was evaluated by repeatability (*n* = 5), resulting in coefficient of variation (CV, %) values below 10% (Table 7). These values indicate that the method is highly precise. Concerning accuracy, recoveries of fucoxanthin and β-carotene were calculated by pre-spiking *H. elongata* MAE extract before the extraction process at three different levels. As displayed in Table 7, recoveries of fucoxanthin were found to be 70.0 ± 0.1% (concentration added: 0.0033 mg/mL), 54.2 ± 0.1% (concentration added: 0.0024 mg/mL), and 74.1 ± 1.5% (concentration added:0.00045 mg/mL). For β-carotene, when the matrix was spiked with 0.0117, 0.0085, and 0.0016 mg/mL, recoveries were 82.9 ± 1.9, 99.5 ± 1.3, and 99.0 ± 1.5%, respectively. Most of these values are within the acceptable range of recovery percentages (70–120%) [10,52]. However, as pointed out by Rodriguez-Amaya [52], recovery tests based on the spiking of samples with standards are questionable for compounds such as carotenoids, which are well protected by membranes and cell walls and can be linked to other components in natural matrices. False recovery percentages can be obtained and should be interpreted with caution because the spiked analytes do not behave in the same way as the endogenous protected compounds.

**Figure 6:**
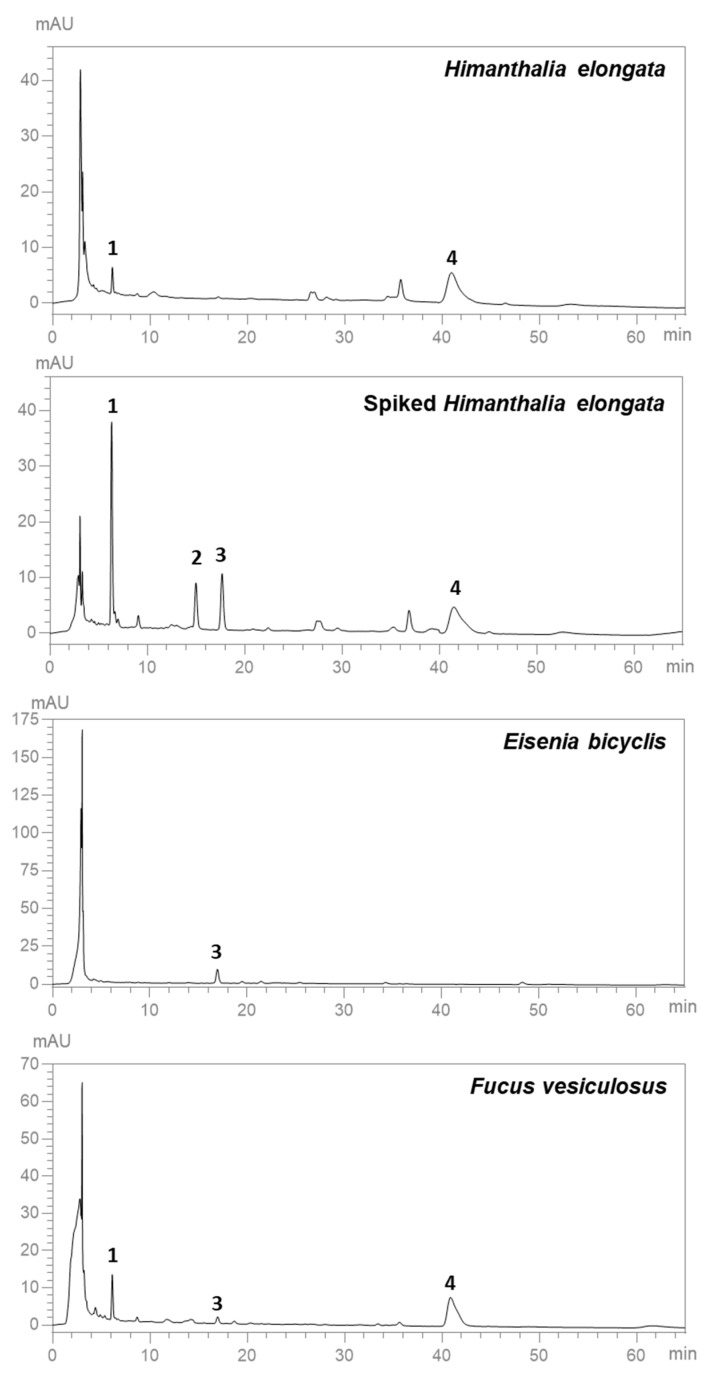
HPLC-DAD chromatograms obtained at 450 nm with the optimized MAE extracts of *H. elongata*, *H. elongata* spiked with the four carotenoid standards, *E. bicyclis*, and *F. vesiculosus*. Identity of the compounds as in Table 6.

Lutein was not detected in any MAE extracts analysed in the HPLC-DAD. On the other hand, fucoxanthin and β-carotene were identified in *H. elongata* and *F. vesiculosus* extracts, while zeaxanthin was detected in *E. bicyclis* and *F. vesiculosus* (Figure 6).

Nonetheless, Kim et al. [53] reported a fucoxanthin content of 0.08–0.26 mg/g fresh algae for *E. bicyclis*. Moreover, a series of chlorophyll derivatives were observed in all extracts, in which their UV/vis spectra showed two bands, one at 401–436 nm and the other at 656–673 nm. Table 8 summarises the content of the three carotenoids (fucoxanthin, zeaxanthin, and β-carotene) in the analysed extracts, achieving 0.0407 µg/mg extract d.w. for *H. elongata*, 0.0845 µg/mg extract d.w. for *E. bicyclis*, and 0.155 µg/mg extract d.w. for *F. vesiculosus*.

Garcia-Perez et al. [54] have recently quantified the amount of chlorophylls, chlorophyll derivatives, and carotenoids, including fucoxanthin, zeaxanthin, and β-carotene, in nine brown algae. Comparing different solvents (ethanol, acetone, ethyl acetate, chloroform, and hexane), they concluded that ethanol and acetone were the most efficient for chlorophyll and carotene extraction, while chloroform is likely to be the best ones for recovering xanthophylls. Concerning *H. elongata*, these authors obtained different amounts of fucoxanthin (1512.0–3114.1 µg/g alga d.w.), zeaxanthin (57.9–119.5 µg/g alga d.w.), and β-carotene (1670.3–6141.7 µg/g alga d.w.) depending on the extraction solvent applied. Indeed, Garcia-Perez et al. [54] found that the amount of fucoxanthin (0.009–18.60 mg/g alga d.w.) and β-carotene (0.0095–0.58 mg/g alga d.w.) reported in the literature for *H. elongata* is quite variable and strongly dependent of the type of extraction and solvent used. Similarly, fucoxanthin content in *F. vesiculosus* also varied from 0.02 mg/g alga d.w. (extracted with ethanol) to 0.699 mg/g alga d.w. (extracted with acetone) [55,56]. Regarding *E. bicyclis*, a value of 0.42 mg fucoxanthin/g d.w. was found in previous work [47], although this xanthophyll was not detected in our sample.

### 3.5. Contribution of Seaweed Consumption to Dietary Carotenoid Intake

Vitamin A (VA) is essential for all vertebrate animal species. It is obtained from the diet either as preformed VA from animal foods or as provitamin A carotenoids, mainly β-carotene, α-carotene, and β-cryptoxanthin, from vegetable foods. Although all carotenoids with one or more unsubstituted β-ionone rings can theoretically be VA precursors, β-carotene appears to be the most relevant. As mentioned, VA is only present in animal products such as liver, eggs, and milk products, therefore in countries where food is scarce or there is a low intake of animal products, carotenoids are essential to meet VA dietary requirements. Indeed, carotenoids contribute to 80% or more in Asian and African countries. Even in developed countries, carotenoids usually contribute to vitamin A supply by more than 40% [57]. In the Western diet, about 20 to 34% of the habitual intake of vitamin A originates from provitamin A carotenoids [58]. In contrast, most individuals in developing countries require >70% of provitamin A carotenoids in the diet [59]. The effect of food matrices of vegetables and fruits in which β-carotene is incorporated has been found to exert a major influence on the measured VA equivalency of β-carotene [60].

There is no established dietary recommendation for carotenoids, and the European Food Safety Authority [61] considered that the existing evidence was insufficient to establish a recommended dietary allowance (RDA) or adequate intake (AI) for β-carotene and other carotenoids. However, in most European countries, a recommended intake of 4.8 mg β-carotene per day is suggested, assuming that this quantity is needed to meet the daily 800 micrograms of vitamin A (conversion factor 6) [61]. However, the conclusions of many epidemiological studies [62] have revealed that a plasma level of 0.4 µmol/L β-carotene should be aimed at regarding its potential health benefits. This concentration can be reached with 2–4 mg/day of β-carotene [62].

Consumption of foods rich in β-carotene is highly recommended since it is associated with a lower risk of chronic diseases and ensures sufficient antioxidants. Therefore, a healthy and varied diet containing 100–500 g/d of fruit and vegetables should provide a high proportion of carotenoid-rich food [62].

Jiang et al. [63] concluded that a higher intake of specific vitamins and carotenoids was associated with a lower risk for age-related cataracts (ARC) (a 10% decreased risk, R.R.: 0.90; 95% CI: 0.83, 0.99; *p* = 0.023). In addition, increased intake of lutein/zeaxanthin (particularly at doses > 10 mg/d) was considered to help maintain ocular health and to be associated with a clinically important change in macular pigment optical density and visual function [64].

Edible seaweeds are a valuable source of natural pigments like carotenoids, chlorophylls, and phycobiliproteins, which are recognized for their advantageous therapeutic effects [65]. However, their interactions affecting bioaccessibility are not yet fully understood. In a recent study [66], eight types of dietary chlorophylls and their derivatives were combined with β-carotene across six different oil types (corn oil, coconut oil, medium-chain triglycerides, peanut oil, olive oil, and fish oil) and subjected to in vitro digestion. Overall, chlorophylls significantly reduced the bioaccessibility of β-carotene by competitively incorporating into micelles [66]. This antagonistic effect may reduce the effective absorption of carotenoids, depending on the specific type and structure of chlorophyll present in different seaweed species. However, studies suggest that chlorophylls and carotenoids may work synergistically to enhance antioxidant effects, providing greater protection against oxidative stress. Chlorophylls and carotenoids when combined can more effectively neutralize free radicals through processes like hydrogen atom transfer, which are essential for cell protection. This interaction also appears to facilitate the reduction of oxidative markers, amplifying the protective benefits each compound offers individually [67].

Nowadays, due to insufficient intake of foods with adequate micronutrient content or bioavailability, micronutrient malnutrition is emerging as a silent epidemic, known as hidden hunger, unseen and uncountable until adverse health effects appear. Several approaches can be considered to increase the apport of micronutrients, namely carotenoids, such as dietary diversification, supplementation, biofortification, and food fortification, but the novel approach is food-to-food fortification. In this technique, micro- or macronutrient-dense foods are added to nutrient-deficient foods to increase their nutritional properties [68,69]. It has a much broader and more sustained impact on providing essential micronutrients to targeted populations [70]. Seaweeds or foods fortified with seaweed extracts can help achieve the daily intake of β-carotene and meet VA dietary requirements. Nonetheless, further research is needed to understand how chlorophylls and carotenoids interact within seaweed matrices, as these interactions may influence the bioaccessibility and effectiveness of seaweed-derived carotenoids in fulfilling vitamin A requirements. The competitive dynamics between β-carotene and various chlorophyll derivatives remain poorly understood, yet clarifying these mechanisms is essential for evaluating their effects on nutrient absorption and overall health benefits [66].

Despite all the benefits attained from seaweed consumption, further studies should also consider organic and inorganic pollutants (heavy metals, polycyclic aromatic hydrocarbons), as well as other essential elements such as iodine that exist at high levels in seaweeds (iodine can be harmful to health if excessively consumed), in order to assess the safety of seaweed consumption [71].

## 4. Conclusions

This study aimed to optimise the extraction of carotenoids from different species of seaweeds using a BBD. Concerning the extraction solvent, 90% ethanol was the most efficient one; besides being a more selective solvent when removing carotenoids in comparison with acetone, it is a product of choice for incorporation into the food industry, not being harmful to human health. RSM analysis also indicated some factors that interfere with the amount of carotenoids extracted. For example, temperature and solid/solvent ratio were critical factors in increasing the extracted amount. These factors were optimised by the statistical tool Stat-ease 11 Design-Expert using a Box–Behnken design, which provided the optimum conditions for obtaining the maximum carotenoid content of the algae under study, correlating the variable factors during the extraction process.

Seaweeds are marine organisms that have been increasingly used for various purposes but with great emphasis on the food industry and cosmetics due to their beneficial properties for the human being, in particular their antioxidant power that affects many metabolic pathways of the human body. One of the most prominent biocompounds of these algae is β-carotene, which, as a precursor of vitamin A, shows promising effects on the eye health of humans. Another important pigment is fucoxanthin, which is a good antioxidant with a great capacity for anti-ageing and anti-tumour activity.

## Figures and Tables

**Table 1:** BBD matrix with three independent variables (X1—time, X2—temperature, and X3—solid/solvent ratio).

Run	X1: Time (min)	X2: Temperature (°C)	X3: Solid/Solvent Ratio (g/mL)
1	5	40	1/15
2	25	60	1/10
3	25	40	1/5
4	15	40	1/10
5	5	20	1/10
6	15	20	1/5
7	15	20	1/15
8	25	40	1/15
9	25	20	1/10
10	15	40	1/10
11	5	60	1/10
12	15	60	1/5
13	15	60	1/15
14	5	40	1/5
15	15	40	1/10

**Table 2:** Microwave conditions for the assay with 1/15 solid/solvent ratio (g/mL).

Test Ratio 1/15; 3 Replicas						
Power		Ramp	PSI	Temperature	Control	
Max	%	(min.)		(°C)	S	Hold
1000 W	100	5:00	150	60	2	15:00 min

**Table 3:** The yield and total carotenoid and chlorophyll content of *H. elongata* under conventional extraction.

Solvent	Solid/Solvent Ratio (g/mL)	Temperature (°C)	Yield (%)	Total Carotenoids (μg/mg Extract d.w.)	Total Chlorophylls (μg/mg Extract d.w.)
100% Ethanol	1/5	25	8.36	0.00502 ± 0.0004 ****	0.0219 ± 0.0018 ****
100% Ethanol	1/10	25	10.1	0.0216 ± 0.0017 ****	0.185 ± 0.015 ****
100% Ethanol	1/15	25	11.7	0.0322 ± 0.0026 ****	0.487 ± 0.039 **
100% Ethanol	1/10	40	13.6	0.0431 ± 0.0034 ****	0.605 ± 0.048 *
100% Ethanol	1/15	40	15.1	0.0450 ± 0.0045 ****	0.638 ± 0.063 *
90% Ethanol	1/10	25	12.9	0.0142 ± 0.0014 ****	0.0653 ± 0.0065 ****
90% Ethanol	1/15	25	12.2	0.0192 ± 0.0017 ****	0.150 ± 0.014 ****
90% Ethanol	1/10	40	16.1	0.0326 ± 0.0029 ****	0.289 ± 0.026 ****
90% Ethanol	1/15	40	15.9	0.0353 ± 0.0032 ****	0.451 ± 0.041 **
50% Ethanol	1/10	25	18.2	0.00317 ± 0.0003 ****	0.0165 ± 0.0015 ****
50% Ethanol	1/15	25	18.7	0.00275 ± 0.0002 ****	0.0101 ± 0.0009 ****
50% Ethanol	1/10	40	20.5	0.00411 ± 0.0003 ****	0.0115 ± 0.0009 ****
50% Ethanol	1/15	40	20.6	0.00613 ± 0.00049 ****	0.0173 ± 0.001384 ****
100% Acetone	1/5	25	10.4	0.0126 ± 0.0010 ****	0.0556 ± 0.0045 ****
100% Acetone	1/10	25	11.3	0.0459 ± 0.0046 ****	0.636 ± 0.0636 *
100% Acetone	1/15	25	11.1	0.0635 ± 0.0044 ****	0.710 ± 0.050 n.s.
100% Acetone	1/10	40	12.1	0.0813 ± 0.0057 **	0.907 ± 0.063 n.s.
100% Acetone	1/15	40	12.2	**0.0942 ± 0.0066**	**1.08 ± 0.08**
90% Acetone	1/10	25	13.1	0.0416 ± 0.0029 ****	0.285 ± 0.020 ****
90% Acetone	1/15	25	12.4	0.0667 ± 0.0060 ****	0.732 ± 0.066 n.s.
90% Acetone	1/10	40	14.3	0.0746 ± 0.0067 ****	0.887 ± 0.80 n.s.
90% Acetone	1/15	40	13.5	0.0609 ± 0.0043 ****	0.592 ± 0.041 *

In the same column, all extraction conditions were compared to the condition that yielded the highest concentration of carotenoids and chlorophylls (indicated in bold). Statistical significance is denoted as follows: (****, *p* < 0.0001; **, *p* < 0.01; *, *p* < 0.05; n.s., *p* > 0.05).

**Table 4:** Carotenoids content obtained from MAE using *H. elongata* in 90% ethanol. Actual and predicted values were obtained with a reduced BBD design, with three central points.

Run	Actual Value (µg Carotenoids/mL)	Predicted Value (µg Carotenoids/mL)	Actual Value (µg/mg Extract d.w.)
1	2.28	2.36	0.0440 ± 0.01176
2	2.30	2.64	0.0570 ± 0.0009
3	0.747	0.857	0.0177 ± 0.0004
4	1.90	2.08	0.0352
5	1.86	1.51	0.0507 ± 0.0131
6	0.511	0.290	0.0176 ± 0.0030
7	1.54	1.79	0.0325 ± 0.0044
8	2.40	2.36	0.0468 ± 0.0064
9	1.47	1.51	0.0345 ± 0.0101
10	2.16	2.08	0.0360
11	2.76	2.64	0.0549 ± 0.0016
12	1.64	1.42	0.0634 ± 0.0288
13	3.21	2.93	0.0619 ± 0.0010
14	0.531	0.857	0.0134 ± 0.0033
15	2.10	2.08	0.0548

**Table 5:** Carotenoid content obtained from MAE using 90% ethanol.

Macroalgae	M_extract_ (mg)	Total Carotenoids (µg/mL)	Total Carotenoids (µg/mg Extract d.w.)
*C. tomentosum*	53.2 ± 5.7	3.76 ± 0.28	0.143 ± 0.023
*F.vesiculosus*	50.8 ± 4.9	3.85 ± 0.23	0.153 ± 0.021
*E. bicyclis*	66.3 ± 3.8	4.20 ± 0.35	0.127 ± 0.017
*H. elongata*	99.5 ± 7.8	2.12 ± 0.17	0.0428 ± 0.0051
*G. gracilis*	31.6 ± 4.6	1.19 ± 0.16	0.0574 ± 0.0157

**Table 6:** Parameters of the calibration curves of carotenoids.

	Compound	RT (min)	[Range] (mg/mL)	Equation	*R* ^2^	LOD (mg/mL)	LOQ (mg/mL)
1	Fucoxanthin	6.13	1.44 × 10^−5^–7.20 × 10^−2^	y = 1.64 × 10^8^x + 7.70 × 10^3^	0.9997	6.78 × 10^−7^	2.05 × 10^−6^
2	Lutein	14.20	9.80 × 10^−6^–4.90 × 10^−2^	y = 1.33 × 10^8^x − 8.61 × 10^0^	0.9998	1.29 × 10^−6^	3.90 × 10^−6^
3	Zeaxanthin	16.96	1.10 × 10^−5^–5.50 × 10^−2^	y = 1.53 × 10^8^x − 8.75 × 10^4^	0.9974	3.47 × 10^−6^	1.05 × 10^−5^
4	β-Carotene	41.00	2.73 × 10^−5^–5.45 × 10^−1^	y = 6.16 × 10^7^x + 4.18 × 10^5^	0.9928	5.68 × 10^−7^	1.72 × 10^−6^

**Table 7:** Intraday and interday (*n* = 5) coefficients of variation (CV, %) and recoveries (%) of fucoxanthin and β-carotene from *H. elongata*.

Compound	Intraday	Interday	Recovery
Fucoxanthin	6.85	8.10	54.2–74.1
β-Carotene	1.84	4.52	82.9–99.5

**Table 8:** Carotenoid content in macroalgae extracts (*n* = 3).

Macroalgae	Fucoxanthin (µg/mg Extract d.w.)	Zeaxanthin (µg/mg Extract d.w.)	β-Carotene (µg/mg Extract d.w.)	Σ (µg/mg Extract d.w)
*Himanthalia elongata*	0.00950 ± 0.00024	n.d.	0.0312 ± 0.00208	0.0407
*Eisenia bicyclis*	n.d.	0.0845 ± 0.0077	n.d.	0.0845
*Fucus vesiculosus*	0.0305 ± 0.0050	0.0943 ± 0.0330	0.0297 ± 0.0041	0.155

## Data Availability

The datasets analysed in the current study are available from the corresponding author on reasonable request.

## Supplementary Materials

The following supporting information can be downloaded at: https://www.mdpi.com/article/10.3390/life14121573/s1, Figure S1. Diagnostic graphics, obtained from Design Expert 11, with a reduced BBD design, with three central points. A-Normal Plot of Residuals; B-Residual vs. Predicted; C- Predicted vs. Actual; D-Residuals vs. Run; E-Residuals vs. B: Temperature; F-Residuals vs. Ratio.
