# Supplementary material for: Sustainable Carotenoid Extraction from Macroalgae: Optimizing Microwave-Assisted Extraction Using Response Surface Methodology
Source: Life (Basel). 2024 Nov 30;14(12):1573. doi: 10.3390/life14121573 (PMC11676899; doi:10.3390/life14121573)
Supplement: Supplementary file 1 [file life-14-01573-s001.zip › life-3286205-supplementary.pdf]

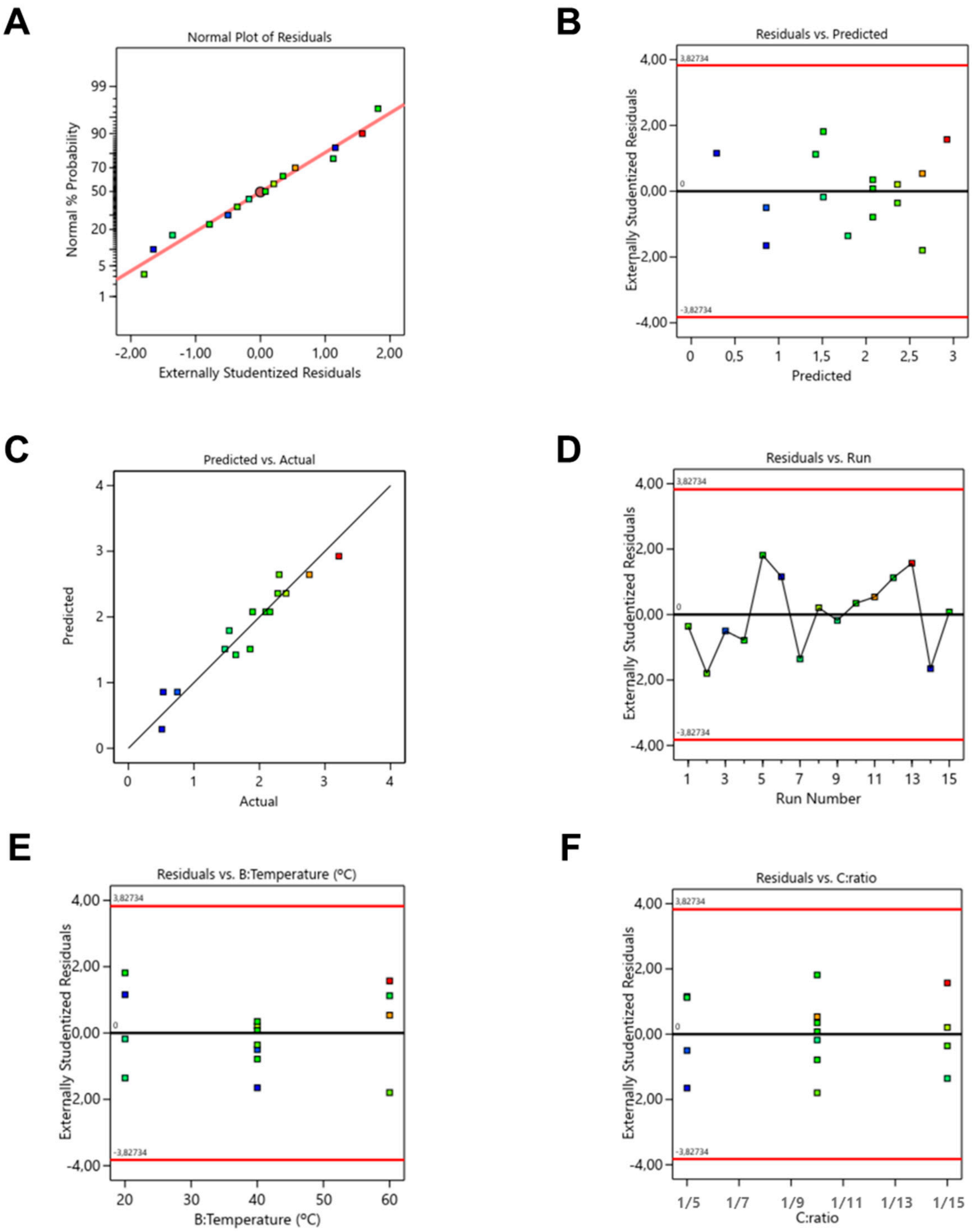

Figure S1. Diagnostic graphics, obtained from Design Expert 11, with a reduced BBD design, with three central points. A-Normal Plot of Residuals; B-Residual vs. Predicted; C- Predicted vs. Actual; D-Residuals vs. Run; E-Residuals vs. B: Temperature; F-Residuals vs. Ratio.
